# ^99m^tc-Ubiquicidin [29–41], a Promising Radiopharmaceutical to Differentiate Orthopedic Implant Infections from Sterile Inflammation

**Published:** 2013

**Authors:** Davood Beiki, Gholamali Yousefi, Babak Fallahi, Mohammad Naghi Tahmasebi, Ali Gholamrezanezhad, Armaghan Fard-Esfahani, Mostafa Erfani, Mohammad Eftekhari

**Affiliations:** a *Research Center for Nuclear Medicine, Tehran University of Medical Sciences, Tehran, Iran.*; b *Department of Orthopedic and Trauma Surgery, Shariati Hospital, Tehran University of Medical Sciences, Tehran, Iran. *; c *Nuclear Science Research School, Nuclear Sciences and Technology Research Institute, Atomic Energy Organization of Iran, Tehran, Iran. *

**Keywords:** Radiopharmaceutical, Ubiquicidin [29-41], Technetium-99m, Scintigraphy, Orthopedic implant, Infection, Inflammation

## Abstract

Ubiquicidin (UBI) [29-41] is a synthetic cationic antimicrobial peptide that preferentially binds to bacterial cell membrane at the site of infection. We aimed to assess diagnostic value of ^99m^Tc-UBI [29-41] as a radiopharmaceutical in differentiation of bacterial infection from sterile inflammation in suspected orthopedic implants. Nine patients suspected for orthopedic implant infection, all males with the mean age of 41.6 ± 20.9 years, were studied. A dose of 10 MBq/Kg (range : 555-740 MBq) ^99m^Tc-UBI [29-41] was injected intravenously. A dynamic study followed by static whole body imaging at 30, 60 and 120 min post-radiotracer injection was acquired. Periprosthetic tissue culture was considered the closest test to a gold standard for diagnosing infections and scintigraphic scans were categorized as true- or false-positive and true- or false-negative, considering the bacterial culture as the gold standard. No adverse reaction was observed during or after the radiotracer injection days. There were five true positive, four true negative and no false positive and false negative scans. Sensitivity, specificity, positive predictive value (PPV) and negative predictive value (NPV) were all calculated as 100%. We found a high diagnostic accuracy for ^99m^Tc-UBI [29-41] scintigraphy in differentiation of bacterial infection from sterile inflammation in suspected orthopedic implants. Therefore, ^99m^Tc-UBI [29-41] scintigraphy might be potentially recommended as a safe and promising imaging modality in these settings. However, further studies on a larger number of patients and different pathologies are still needed.

## Introduction

As the number of orthopedic procedures continues to rise, there is a substantial increase in surgical implantation of internal devices and prostheses. Accordingly, the diagnosis of orthopedic implant infections, as one of the most important complications of the procedure, is becoming an increasingly common challenge. Oftentimes, the non-invasive diagnostic differentiation between infection and sterile inflammation is problematic, while clinical management decisions need to be made promptly in order to avoid subsequent serious complications. The current available imaging approaches include ultrasound (1, 2), computed tomography (CT) (3, 4), magnetic resonance imaging (MRI) (5, 6), ^99m^Tc bone and/or Gallium citrate scintigraphy (7, 8), labeled antigranulocyte antibody or labeled leukocyte study (9), labeled antibiotic such as ciprofloxacin (10), the avidin-biotin system imaging (11-13) and FDG-PET (14-17), which despite the high sensitivity, lack specificity for infections (18, 19). Therefore, advances in the non-invasive differentiation between infection and sterile inflammation are needed.

Ubiquicidin (UBI) [29-41] is a synthetic cationic antimicrobial peptide that preferentially binds to bacterial cell membrane at the site of infection (19-25). Considering its affinity for bacterial components, UBI [29-41] has been labeled with ^99m^Tc and tested as a potential scintigraphic agent for diagnosis of suspected orthopedic implant infections (26). Limited, but promising, preclinical and pilot studies on this radiopharmaceutical were recently published, but they suffer from remarkable shortcomings, the main one being the absence of a reliable gold standard; therefore, to provide multicentral evidence on its efficacy, further studies were encouraged (26). We aimed to assess the diagnostic value of ^99m^Tc-UBI [29-41] scintigraphy in differentiation of bacterial infection from sterile inflammation in suspected orthopedic implants.

## Experimental

*Radiopharmaceutical*

^99m^Tc-Pertechnetate fresh elute from the ^99^mo/^99m^Tc Generator was used to label the antimicrobial peptide UBI [29-41], which both were supplied by the Radioisotope Division, Atomic Energy Organization of Iran. The radiopharmaceutical was prepared by adding ^99m^Tc-pertechnetate fresh elute to HYNIC-UBI [29-41] kit and incubating for 15 min at room temperature. The radiopharmaceutical kit was stored at room temperature and used within 6 h of reconstitution. All the labeling and quality controls were done according to the manufacturer’s instructions. 

*Patients*

Fourteen consecutive patients, suspicious for implant infection with no history of antibiotic therapy for their present condition, were enrolled in the study. In addition, patients with known hepatic and/or renal disease or history of hypersensitivity state were excluded from the study. The study was approved by the committee on ethics, Tehran University of Medical Sciences and each subject gave written informed consent.

*Procedure*

Imaging procedure was done with patients in supine position, using a Dual head gamma camera (ADAC, USA) equipped with LEGP collimator. Immediately after the intravenous injection of 10 MBq/Kg (range: 555-740 MBq) of ^99m^Tc-UBI [29-41], dynamic imaging with 10 sec for each frame up to 10 min post-injection was acquired from the target and the contralateral body regions. Besides, the whole-body anterior and posterior views were obtained at 30, 60, and 120 min after the injection and scanning speed was set at 12 cm/min in all acquisition procedures. The SPECT images were obtained at 2 h post-injection, if needed. The photopeak was set at 140 KeV for ^99m^Tc.

Visual scoring system was used to categorize studies as positive or negative. The scans were graded as follows (27): 0 (minimal or no uptake; less than or equivalent to soft tissue); 1 (mild; target uptake less than the liver activity); 2 (moderate; uptake greater than or equal to that of the liver activity) and 3 (intense; uptake greater than or equal to that of the kidneys). Lesion activities with grades of 0 or 1 were considered as negative scan and those with grades of 2 or 3 considered as positive scan. All scintigraphic images were interpreted by two experienced nuclear medicine physicians blinded to all radiological and laboratory data and the final decision was made by consensus.

As there is no definite non-invasive gold standard for detection of orthopedic implant infection, periprosthetic tissue culture was considered as the closest test to a gold standard for diagnosing infections and scintigraphic scans were categorized as true- or false-positive and true- or false-negative, considering the bacterial culture as the gold standard. For this purpose, samples for culturing were taken from the site by an experienced orthopedist.

*Statistical analysis*

Sensitivity (proportion of true positives identified correctly by the scintigraphy), specificity (proportion of true negatives identified correctly by the scintigraphy), positive predictive value (PPV: proportion of patients with infection who were diagnosed correctly) and negative predictive value (NPV: proportion of patients with negative test results who are diagnosed correctly) of^ 99m^Tc-UBI [29-41] scintigraphy were calculated.

## Results and Discussion

Fourteen patients underwent ^99m^Tc-UBI [29-41] scintigraphy. As five patients refused the tissue sampling procedure, nine patients, all males with the mean age of 41.6 20.9 years, were included in the final analysis. Three objects had hip prosthesis, four internal fixators of tibia, one internal fixator of femur and one internal fixator of humerus. Based on the results of bacteriological culture, five patients had orthopedic implant infections, the pathogenic microorganism in all of which was *Staphylococcus aureus*.

No adverse reaction was observed during or after the radiotracer injection days. In six patients, the scan was interpreted as positive and in three of them, as negative. In all positive studies, the radiotracer activity at the implant site was higher than adjacent tissues in early images (positive blood pool phase). No significant difference was seen in the intensity of uptake in scans between the 30, 60 and 120 min images. Considering the bacteriologic culture as the gold standard, there were five true positive, four true negative and no false positive and false negative scans. Sensitivity, specificity, PPV, NPV and diagnostic accuracy were all calculated as 100%.

Currently available non-invasive imaging modalities suffer from remarkable limitations in the assessment of inflammatory diseases involving internal devices and prostheses. For example, bone scintigraphy, Gallium scans, and ^18^F-FDG PET, in spite of their high sensitivity, offer poor sensitivity and MRI is limited due to the artifacts induced by metallic implants (18, 28, 29). Regarding these limitations, a need for an alternative imaging approach has been emphasized.

*In-vitro *studies have shown a specific binding of ^99m^Tc-labeled UBI to bacteria and it has been suggested that the accumulation at infection sites, could be the result of its high thermodynamic stability, selectivity and stereo specificity (19). On the other hand, *in-vivo *studies have revealed that there is a significant difference in the ^99m^Tc-UBI uptake between the bacterial infection and non-bacterial inflammation sites compared to ^67^Ga-citrate, with an average infection/inflammation ratio of 2.08 ± 0.49 for ^99m^Tc-UBI and 1.14 ± 0.45 for^ 67^Ga-citrate (25). These findings prompted investigators to apply ^99m^Tc-UBI [29-41] scintigraphy for the diagnosis of various human infectious processes and to differentiate it from sterile inflammation (18, 30, 31).

We found an excellent diagnostic accuracy for ^99m^Tc-UBI [29-41] scintigraphy, which supports the promising previous reports (Table 1).

**Table 1:** A review on clinical trials assessing the accuracy of ^99m^Tc-UBI [29-41] scintigraphy in various musculoskeletal applications.

**Author/Year**	**Sample size**	**Pathology**	**Sensitivity**	**Specificity**	**Accuracy**	**Gold standard**
Assadi et al. 2011 (1)	20	11 Diabetic ulcer, 5 fracture or orthopedic implant, 4 miscellaneous infections	100	100	100	consensus of clinicians considering clinical and paraclinical data
Meléndez-Alafort et al. 2004 (7)	6	Pediatric cases suspicious for osteomyelitis cases	100	100	100	Gallium Scintigraphy
Dillmann-Arroyo et al. 2011 (13)	27	Vertebral osteomyelitis (12 with orthopedic implants)	100	88	-	histopathologic study or microbiologic culture or with the clinical findings after a follow-up of > 6 months
Akhtar et al. 2005 (10)	18	10 soft-tissue infections , 3 bone infection, 1 patient with no bacterial infection	100	80	94.4	bacterial culture as the major criterion and clinical tests, radiography, and 3-phase bone scanning as minor criteria
Cumulative value	71	-	100	90.4	-	-

 These studies altogether suggest the application of ^99m^Tc-UBI [29-41] as a reliable imaging modality for differentiation of bacterial infection from sterile inflammation in suspected orthopedic implants. Although the role of this method in diagnostic management of patients with suspected orthopedic implant infection has yet to be defined in larger number of patients and multicentral studies, the approach seems to be promising to arrive at a reliable and early non-invasive diagnosis. Previously, the potential role of quantitative ^99m^Tc-UBI [29-41] scintigraphy to monitor antibiotic therapy in patients with orthopedic infection has been suggested, as significant reduction in radiotracer uptake after a successful treatment is seen (26). In studies by Nibbering *et al.*, ^99m^Tc-UBI [29-41] scintigraphy showed an inverse correlation between intensity of radiopharmaceutical uptake and dose of antibiotic in the infection focus (32, 33), which further suggest its application for treatment monitoring purposes.

To date, more than 70 patients suspicious for osteomyelitis and orthopedic implant infection have been studied with ^99m^Tc-UBI [29-41] scintigraphy, which has resulted in accuracy indices of more than 80% in the published reports (Table 1). However, application of ^99m^Tc-UBI [29-41] scintigraphy is not limited to musculoskeletal indications. Vallejo *et al. *have applied the same imaging technique to diagnose the mediastinitis after cardiac surgery and reported a high diagnostic accuracy of more than 90% (34). Sepulveda-Mendez *et al. *also found a specificity of 95.35%, sensitivity of 97.52%, positive predictive value of 96.72%, negative predictive value of 96.47%, and the accuracy of 96.62% for ^99m^Tc-UBI [29-41] scintigraphy in 196 patients with fever of unknown origin (35). Brouwer *et al*. suggested that ^99m^Tc-UBI [29-41] scintigraphy can be a dedicated non-invasive imaging tool for the early detection of infective endocarditis (36).

*Technical considerations*

**Figure 1:**
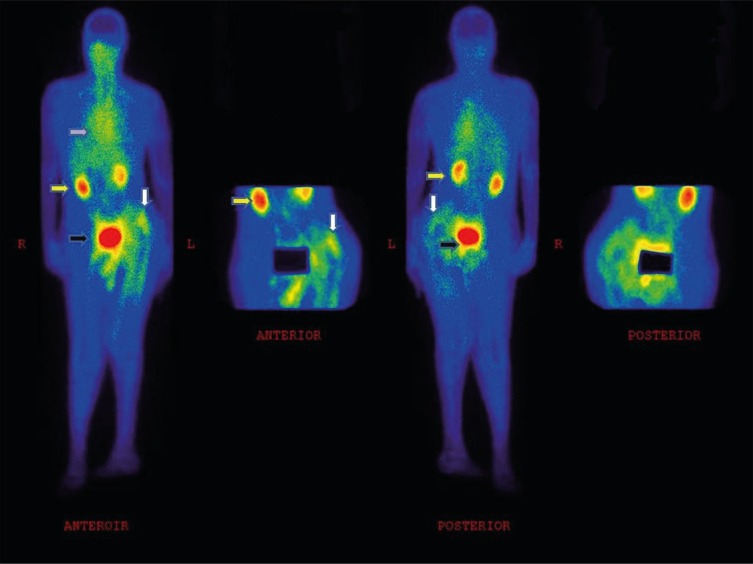
A 62 y/o patient (weight: 74 Kg) with left hip prosthesis, showing increased ^99m^Tc-UBI [29-41] uptake corresponding to the region of prosthesis. The arrow in white shows the infection site, the arrow in yellow shows radiotracer excretion by the kidneys, the arrow in black shows radiotracer accumulation in bladder, and the arrow in purple shows cardiac blood pool activity.

In our study, we found no significant difference in the intensity of radiopharmaceutical uptake between 30, 60 and 120 min images. This finding is supported by previous studies (18, 20, 37) and could be considered as an indirect evidence of the strong radiopharmaceutical avidity for the target peptide. It also suggests that ^99m^Tc-UBI [29-41] will be cleared rapidly from the circulation with a first pass-like pattern (18, 27) and a high target to background ratio is achieved as early as 15 min post-injection. The radiopharmaceutical shows fast renal clearance with negligible liver uptake and hepatobiliary excretion (Figure 1). Therefore, ^99m^Tc-UBI [29-41] scintigraphy can be completed in just half an hour after the injection, as delayed imaging adds no additional finding to the study. It has been confirmed that the effective dose is within acceptable range, even for application in pediatric population (24).

*Safety profile*

No adverse reaction was seen in our population, which is in accordance with the previous reports (18, 27, 38). The safety profile and lack of hazards of handling blood products (the major disadvantage of labeled leukocytes) as well as its applicability to leukopenic patients and low probability of resistance to antimicrobial peptides has been considered as the major advantages of ^99m^Tc-UBI [29-41] scintigraphy (17).

*Study limitation*

Small sample size of the study was the major limitation. Besides, in our study, all patients with infected implants had *Staphylococcus aureus *positive cultures, which are explained by the fact that *Staphylococcus aureus *is the most common cause of osteomyelitis in this setting. Akhtar *et al. *reported that ^99m^Tc-UBI [29-41] shows less avidity at sites infected with E. coli than S. aureus and concluded that the lower accumulation with E. coli might be explained by either the lower virulence of the organism or diminished affinity of the peptide for E. coli membranes (39). Larger study populations probably will increase a higher probability of covering other pathogenic organisms and will provide the opportunity to assess the diagnostic accuracy of ^99m^Tc-UBI [29-41] scintigraphy in other pathogenic organisms.

## Conclusion

We found a high diagnostic accuracy for ^99m^Tc-UBI [29-41] scintigraphy in differentiation of bacterial infection from sterile inflammation in suspected orthopedic implants. Therefore, ^99m^Tc-UBI [29-41] scintigraphy might be potentially recommended as a safe and promising imaging modality in these settings. However, further studies on larger number of patients and different pathologies are still needed.
